# Advanced Fiber-Coupled Diode Laser Sensor for Calibration-Free 1*f*-WMS Determination of an Absorption Line Intensity

**DOI:** 10.3390/s20216286

**Published:** 2020-11-04

**Authors:** Vladimir Liger, Vladimir Mironenko, Yury Kuritsyn, Mikhail Bolshov

**Affiliations:** Institute of Spectroscopy, Russian Academy of Sciences, 5 Fizicheskaya Str., Troitsk, Moscow 108840, Russia; liger@isan.troitsk.ru (V.L.); miron@isan.troitsk.ru (V.M.); kuritsyn@isan.troitsk.ru (Y.K.)

**Keywords:** diode laser absorption spectroscopy, calibration-free, wavelength modulation, first harmonic, retroreflector, logarithmic processing

## Abstract

A new scheme for a calibration-free diode laser absorption spectroscopy (DLAS) sensor for measuring the parameters of harsh zones is proposed. The key element of the scheme is a micro-prism retroreflector (MPRR). The MPRR facilitates an increase in the mechanical stability of the sensor and a decrease in the background thermal radiation in the hot areas of a tested zone. Reduction in the broadband thermal emission allowed the application of a differential logarithmic conversion (LC) technique for elimination of the residual amplitude modulation and other sources of non-selective attenuation of the probing laser beam. LC allows the use of a 1*f*-wavelength modulation spectroscopy (WMS) detection scheme. Combination of LC and a 1*f*-WMS algorithm provided a new modification of calibration-free DLAS, which could be particularly useful for probing harsh zones with pronounced strong turbulence and high levels of acoustic and electrical noise. The influence of the experimental parameters and characteristics of the main electronic components of the recording and processing system on the accuracy of the integral line intensity determination is investigated theoretically and experimentally. The proposed optical scheme of a DLAS sensor and algorithm for the data processing allowed the integral intensity of an absorption line to be obtained. The potential for the scheme was exemplified with a single water vapor absorption line at 7185.6 cm^−1^. Simultaneous detection of several absorption lines and data processing using the developed algorithm provides the final goal of a DLAS sensor—determination of temperature and partial pressure of a test molecule in a probed gas volume. The developed scheme allows the spatial multiplexing of the radiation of different diode lasers (DLs), which can be used if various test molecules are to be detected, or absorption lines of a test molecule are detected over different wavelength intervals.

## 1. Introduction

Tunable diode laser (DL) absorption spectroscopy (DLAS) is a widely used technique for contactless diagnostics of various gaseous media. DLAS sensors are routinely used for the detection of important air components H_2_O, CO, CO_2_, CH_4_, etc. [1,2,3,4,5,6,7], and are commonly used for measuring temperature and total and partial pressures in hot zones [8,9,10,11]. This technique provides remote, non-perturbing measurements of the parameters of the gaseous media with a time resolution in the micro- to millisecond range depending on the specific experimental conditions. The basic advantages of DLAS sensors are: relatively simple construction, the relatively low cost of the commercial components of the sensor, and the possibility of locating the sensitive part of the sensor away from the hot and/or harsh zone using optical fibers to deliver the probing laser beams to the testing zone. Lastly, highly experienced operators are not necessarily required. Different schemes of DLAS can be used—direct absorption spectroscopy (DAS) and various versions of wavelength modulation spectroscopy (WMS). The temperature of a medium is determined by the ratio of the integral intensities of the absorption lines of a test molecule with different lower energy levels. Knowing the gas temperature, the concentration of a particular molecule is determined by measuring the absorbed intensity of the probing laser beam across the test zone within the media.

Technically, the easiest and most straightforward version of DLAS is scanned-wavelength DAS [12,13,14,15]. This technique does not require calibration to estimate the concentration of the test molecules. The choice of the DAS technique is justified in the case of relatively high signal-to-noise ratios (SNR), when variations of the zero-absorption baseline (BL) cause no serious problems in evaluating the actual line intensities. In the opposite case, with weak lines the correct evaluation of the fluctuating BL, which strongly affects the integral intensity of a weak absorption line, is problematic. The turbulence in a probed media, fluctuations of the laser intensity, and broadband radiation of the hot gas are also serious problems in DAS.

Efficient reduction of the noise problem is provided by the WMS technique [16,17,18,19,20,21]. In scanned-WMS, besides wavelength scanning across the line profile of a test molecule, the wavelength of the DL is modulated with a frequency *f* in the 10–100 kHz range and the transmitted intensity is detected on the harmonics *nf* of the modulation frequency. Elimination of the low-frequency flicker noise increases the SNR and, thus, increases the sensitivity of WMS compared with DAS. This is essential for the diagnostics of turbulent hot zones, such as engine exhausts, shock wave tubes, combustion in gas flows, etc. The drawback of initial versions of the WMS technique is the complex data processing and the necessity for in situ calibration of the sensor.

To overcome this problem, a calibration-free WMS technique (CF-WMS) has been developed, which does not require reference measurements of the laser’s optical intensity [22,23,24,25,26]. Normalization of the absorption signal harmonics *nf*/1*f* (typically 2*f*/1*f*) is most frequently used in CF-WMS. Combination of this normalization with the signal processing, based on the predetermined laser-tuning characteristics and known spectroscopic data, makes it possible to eliminate most calibration factors and infer the temperature and concentration without comparison with measurements in a known gas mixture under known conditions.

A new approach based on the 1*f*-phase detection technique (WMS-*θ*_1*f*_) has been developed [23,27,28,29]. This approach enjoys the benefits of WMS 2*f*/1*f* of providing improvement to the measurement accuracy. However, both versions of WMS need the precise preliminary determination of the tuning parameters and modulation characteristics of the diode laser that is used. Additionally, the raw data processing of (WMS-*θ*_1*f*_) signals is far more complicated compared with DAS.

A sensor based on detection of the first harmonic of the absorption signal has some benefits compared with the detecting of higher harmonics of the systems. The sampling rate required for the 1*f* measurement is half of that required for the corresponding 2*f* measurement. In addition, 2*f*/1*f* detection needs greater memory and speed of the processing unit. The peak-to-peak amplitude of 1*f* is also higher than 2*f*.

One of the radical ways to eliminate the dependence of an absorption signal on the non-selective fluctuations of the laser intensity is a double beam scheme with logarithmic conversion (LC). Several applications of the LC combined with WMS DLAS have been published: the LC in a double beam scheme results in reduction in the excess noise of the laser radiation [30,31]; a large linear dynamic range of the output signal of a sensor using the LC was demonstrated [32]; detection of CH_4_ using LC WMS was shown in [33]. In all cited papers the detection of the second harmonic of relatively low modulation frequency was used. A version of calibration-free WMS based on the combination of WMS and logarithmic processing of the absorption signal is described in [34]. However, so far this combination has only been applied in “purely” laboratory experiments with specially designed absorption cells.

In this paper, we propose an advanced calibration-free version of WMS with enhanced optical depth linearity and better tolerance to the turbulences in the optical path and DL intensity variations. The idea of the technique is based on: (i) the use of a micro-prism retro reflector (MPRR), which provides efficient autofocusing of the transmitted laser beam into the optical fiber; (ii) logarithmic processing of the photodetector signal; and (iii) inference of the absorbance on the selected test molecule absorption lines from the first harmonic of the WMS signal. The key element of the proposed technique is the MPRR, which reflects the laser beam transmitted through the test object back to the multimode fiber through which the beam was delivered to the object. Reflection of the probing laser beam and it’s focusing into the fiber occur automatically. As an additional benefit of MPRR, the optical path of the probing beam inside the test object is doubled.

The length of the fiber can be long enough to separate the sensitive parts of the sensor from the harsh zone, and in this way reduce the electrical pick-up and acoustic noise. A small angle aperture of the fiber and cut-off optical filter efficiently reduce the broadband thermal radiation of a hot object, which allows the logarithmic processing of the photodetector signal, and in this way conversion of the multiplicative components of the photocurrent into the additive ones. The same procedure can be used in the reference channel. The subtraction of the signal in the reference channel of the dual beam scheme from the signal in the signal channel practically eliminates the amplitude modulation of the absorption signal completely, and allows detection of the absorption signal on the 1*f* frequency of WMS.

The modulation frequency should be higher than the frequency of the noise, enabling the signal components to be discriminated through filtration and lock-in detection. The proposed version is much simpler with respect to the processing of the absorption signal as compared with classical calibration-free 2*f/*1*f* WMS and the advanced 1*f*-phase detection technique (WMS-*θ*_1*f*_). The output signal is proportional to the absorbance over a wide range of optical densities and is weakly dependent on the high harmonics of DL intensity. 

To simplify the understanding of the following theoretical background of the proposed version of the sensor, we will start with a short description of the experimental setup. 

## 2. Setup

**Figure 1:**
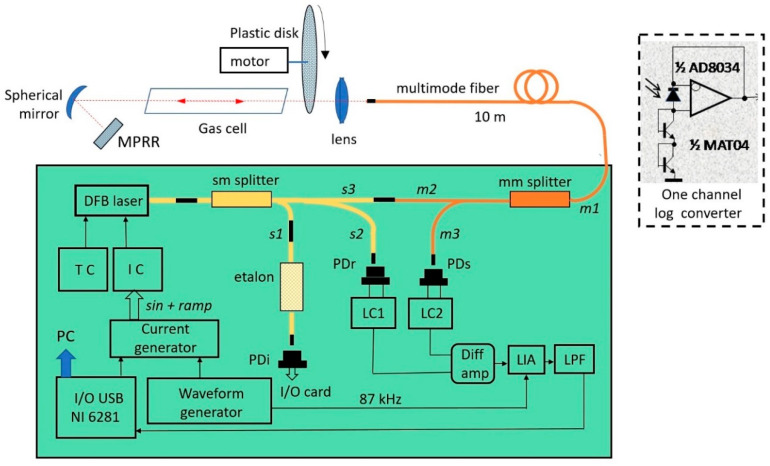
Experimental setup. MPRR—micro-prism retroreflector; TC, IC—temperature and current controllers, respectively; sm—single mode, mm—multimode; *s1*, *s2*, *s3*—single mode fibers; *m1*, *m2*, *m3*—multimode fibers; PDr, PDs, PDi—photodiodes of the reference, sample, and interferometric channels, respectively; LC1, LC2—logarithmic converters of the reference and sample channels, respectively; LIA—lock-in amplifier; LPF—low-pass filter; PC—personal computer. The electric scheme of the LC is shown in the subpanel.

Figure 1 shows a scheme of the experimental setup used for the method validation. A fiber-coupled DFB-laser (NEL709042) operating at around 1.39 μm was used to target water molecule absorption transition, centered at 7185.6 cm^−1^. The laser temperature was stabilized with a Thorlabs TED350 controller. The laser current was tuned by a homemade controller based on a Thorlabs LDC 202 laser driver and an Instek GFG-8219a functional generator. Sinusoidal (*f* = 87 kHz) and ramp signals (122 Hz) were combined to modulate and scan the laser wavelength across the absorption line.

The maximum laser output power was about 10 mW. The laser beam was divided into three parts by a single mode 3-output optical fiber splitter (sm splitter). The first splitter output *s1* (3%) was connected with the fiber ring resonator serving as the wavenumber calibration interferometer (etalon). The second output *s2* (7%) was guided onto the reference photodiode PDr. The third output *s3* (90%) was attached to the input/output *m2* of the multimode (50 µm core) fiber optic splitter (50/50). The light from the multimode splitter (mm splitter) output *m1* after passing along a 10 m long multimode patch cord was collimated by the objective based on a Thorlabs F240APC-C optic collimator and passed through the gas cell. This beam was then reflected from the MPRR (3M diamond-grade microprismatic reflective sheeting 4090) and focused back onto the fiber patch cord by the same objective. Quartz tubes with mica windows were used as the gas cell. The lengths of the tubes varied from 20 cm to 2 m. The cell was filled with the laboratory air at atmospheric pressure. The use of the cells allowed a reduction in the fluctuations of water concentration during the experiments. 

After passing through the multimode splitter in the reverse direction, one part of the reflected beam was guided through the fiber *m3* onto the sample channel photodiode PDs. No focusing optics were used here. The remaining part of the reflected beam can penetrate back to the laser through an FC/APC connector. Such optical feedback can violate normal laser operation, but in our experiments reverse beam intensity was significantly reduced by low light collection efficiency and the large difference in core diameters of the multimode and single mode fibers (50 and 9 µm, respectively). Measured feedback light intensity was less than 0.1%. In principle, introduction of an optical isolator into the scheme can minimize possible optical feedback.

The electronic part of the sensor consisted of simple homemade differential logarithmic converters (LCr, LCs), an analog lock-in amplifier (LIA) and an input/output data acquisition card. Sample and reference channel photodiodes were bootstrapped by an operational amplifier (Analog Devices AD8034). Such a connection reduces effective photodiode capacity and its leakage currents and isolates the differential amplifier from the input of the LC logarithmic elements (subpanel in Figure 1). Transistors from an Analog Devices MAT-04 array connected as diodes were used for logarithmic conversion. The amplified differential signal was demodulated by a homemade LIA based on an Analog Devices AD633 analog multiplier to recover the 1*f* harmonic component, and was digitized via a National Instruments NI USB-6281 data acquisition and processing (DAQ) system. Selection of low-noise components of the input cascades of the electronic detection scheme and zero-bias scheme of the photodiode drastically reduced the noise.

Experiments were performed to validate the sensor characteristics using a static gas cell and an additional scattering unit that simulates high-speed optical perturbations. The scattering unit was made of a plastic disk with a thickness of 100 µm, diameter of about 15 cm, and had a rough surface. With the help of a DC motor, the disk could be rotated at various speeds of up to 3000 rpm perturbing the transmittance of the DL beam and, hence, resulted in random variations of the signal photocurrent.

## 3. Theoretical Background

### 3.1. 1f-WMS and Logarithmic Processing

Assuming linear dependence of the photodiode current on the DL radiation intensity, a general expression for the photodiode current in the signal channel can be written in the form:

(1) $$i_{s}=G_{s}\left[I_{0}\left(t\right)\cdot \tau_{s}\left(t\right)\cdot \tau_{\nu s}\left(t\right)\cdot \exp\left(-\alpha \right)+E\left(t\right)\right]+i_{l}+i_{n}+i_{em}$$

where *G_s_* is the gain of the signal detector, *I*_0_*(t)* is the DL intensity, $\tau_{s}\left(t\right)$ is non-selective transmittance in the signal channel, $\tau_{\nu s}\left(t\right)$ is the selective non-absorbing transmittance defined mainly by interference effects, *E*(*t*) is the broadband background emission intensity, *i_l_* is the leakage current of the photodetector and of the first input channel of the pre-amplifier, *i_em_* is the current defined by electromagnetic noises, *i_n_* is the current defined by the noise of other types, and *α* is the absorbance. From the Beer–Lambert law for a single absorption transition:

(2a) α(ν)=S(T)g(ν) NL

(2b) $$A\;=\;\int^{\;}\alpha \left(\nu \right)d\nu$$

where *S*(*T*) (cm/molecule) is the line strength of selected absorption transition, *N* is the concentration of the absorbing molecule, *L* is the optical path length, and *g*(*ν*) is the frequency-dependent normalized line-shape of the absorption transition. *S*(*T*) is a function of temperature and molecular parameters, and can be calculated as follows:

(3) $$S\left(T\right)\approx S\left(T_{0}\right)\frac{Q\left(T_{0}\right)}{Q\left(T\right)}exp\left[-\frac{hcE^{\prime \prime }}{k}\left(\frac{1}{T}-\frac{1}{T_{0}}\right)\right]$$

where $T_{0}$ is the reference temperature, Q(T) is the partition function of the absorbing molecule, h is Planck’s constant, *c* is the speed of light, $E^{\prime \prime }\;$is the lower-state energy of the transition, and *k* is the Boltzmann constant. *A* (Equation (2b)) is the integrated absorbance (integral line intensity). Almost all terms in Equation (1) depend on time.

DL intensity varies with time due to the linear scan of the DL across the absorption line and WMS modulation of the current. Generally, frequencies range from parts-of Hz to kHz for the wavelength scanning, while hundreds of kHz are used for the WMS mode. Non-selective transmittance $\tau_{s}\left(t\right)$ is defined by the losses in the fiber connectors and multiplexers, scattering on the defects in the optical path, and on small soot particles in the combustion zone. Selective non-absorbing attenuation of the probing DL beam may be caused by reflections on the optical surfaces. Most of these losses are non-stable over time and fluctuate. 

Broadband emission dominates in the combustion zones in the case of incomplete burning and high concentration of soot particles at temperatures above 1000 K. This source of background emission also fluctuates with time.

Photodetector leakage current depends on the type of photodiode and on the off-set voltage. For a fixed electrical circuit, this current and the noise current of the first channel of the pre-amplifier are stable during the measurement cycle. The origin of the electro-magnetic noise could be the nearby high-voltage sources of the experimental propulsion [35]. 

All of these sources of noise affect the precision of the integral line intensity determination. The frequency spectrum of these noises is predominantly a flicker in nature and occupies the band up to several tens of kHz. The spectrum for the electro-magnetic noise can be even broader. The specific construction of the developed sensor minimizes the above sources of noise, reducing Equation (1) to the form:

(4) $$i_{s}=G_{s}\left[I_{0}\left(t\right)\cdot \tau_{s}\left(t\right)\cdot \tau_{\nu s}\left(t\right)\cdot \exp\left(-\alpha \right)\right]$$

A detailed description of the developed construction that provides a drastic reduction in most of the sources of noise will be given in detail in Section 4. 

Following the same logic, and assuming the noise and leakage in the reference channel to be negligible, the current in the reference channel *i_r_* can be written in the form:

(5) $$i_{r}=G_{r}\cdot \tau_{r}\cdot \tau_{\nu r}\cdot I_{0}$$

where *G_r_* is the gain of the diode in the reference channel, and $\tau_{r}$ and $\tau_{\nu r}\;$are the non-selective and selective non-absorbing transmittance in the reference channel, respectively. 

If the DL current is modulated with the frequency *f*, then the laser intensity$\;I_{0}\left(t\right)\;$and instantaneous laser frequency *ν*(*t*) can be described as:

(6) $$I_{0}\left(t\right)=I_{slow}+a\cdot \cos\left(2\pi ft+\psi_{1}\right)+b\cdot \cos\left(4\pi ft+\psi_{2}\right)$$

(7) $$\nu \;=\nu_{slow}+a_{m}\cdot \cos\left(2\pi ft\right)$$

where the subscript “slow” defines the values (*I* or *ν*), which vary slowly during the scanning of the DL frequency; *a* is the laser intensity modulation amplitude (linear part); *b* describes the non-linearity of laser intensity modulation; *ψ*_1_ and *ψ*_2_ are the phase shifts between the modulation of the optical frequency and modulation of laser intensity for the first and second harmonics, respectively; and *a_m_* is the optical frequency modulation amplitude.

If the currents of both signal and reference channels are transmitted through the logarithmic converter (LC), the multiplicative components of the currents will be converted into the additive ones and the output voltage in the signal channel of the ideal LC is:

(8a) $$U_{s}=K\left\{\ln G_{s}+\ln\tau_{s}+\ln\tau_{\nu s}+\ln\left[I_{slow}+a\cos\left(2\pi ft+\psi_{1}\right)+b\cos\left(4\pi ft+\psi_{2}\right)\right]-\alpha \right\}\;$$

and in the reference channel:

(8b) $$U_{r}=K\left\{\ln G_{r}+\ln\tau_{r}+\ln\tau_{\nu r}+\ln[I_{slow}+a\cos\left(2\pi ft+\psi_{1}\right)+b\cos\left(4\pi ft+\psi_{2}\right)]\right\}$$

where *K* is the coefficient of the logarithmic conversion of the currents into the voltages. For the LC based on a *p*-*n* junction, *K* = *kT_pn_*/*q*, where *k* is the Boltzmann constant, *T_pn_* is the temperature of the *p*-*n* junction, and *q* is the electron charge.

Subtracting Equation (8b) from (8a), one obtains the output voltage of the differential logarithmic signal in the form:

(9) $$U_{out}=K\left(\ln\frac{G_{r}}{G_{s}}+\ln\frac{\tau_{r}}{\tau_{s}}+\ln\frac{\tau_{\nu r}}{\tau_{\nu s}}\right)+K\alpha$$

It is important that the differential logarithmic signal does not depend on DL intensity. Due to this, the output signal *U_out_* (Equation (9)) does not contain the laser intensity variations and Residual Amplitude Modulation (RAM) on the modulation frequency *f* during the wavelength scanning.

Two terms in Equation (9) depend on the laser modulation frequency—absorbance *α* and the term accounting for the difference in selective non-absorbing transmittance in the sample ($\tau_{\nu s}$) and reference ($\tau_{\nu r}$) channels. The absorbance *α* can be expanded in the Fourier series in the form:

(10) $$\alpha \left(\nu \right)=\alpha \left(\nu_{slow}+a_{m}\cdot \cos\left(2\pi ft\right)\right)=\overset{\infty }{\underset{0}{\sum }}H_{n}\cdot \cos\left(2\pi nft\right)$$

The coefficients of the harmonics *H_n_* can be calculated according to the expressions:

(11a) $$H_{0}\left(\nu \right)=\frac{1}{2\pi }\int_{-\pi }^{\pi }\alpha (\nu_{\mathrm{slow}}+a_{m}\cos\varphi )d\varphi$$

(11b) $$H_{n}\left(\nu \right)=\frac{1}{\pi }\int_{-\pi }^{\pi }\alpha (\nu_{\mathrm{slow}}+a_{m}\cos\varphi )\cos\left(n\varphi \right)d\varphi$$

Then, the differential voltage of the first harmonic is:

(12) $$U_{out,1f}\left(\nu \right)=K\left[\ln{\left(\frac{\tau_{r}}{\tau_{s}}\right)}_{1f}+\ln{\left(\frac{\tau_{\nu r}}{\tau_{\nu s}}\right)}_{1f}\right]+KH_{1}\left(\nu \right)$$

The first two terms in Equation (12) represent the baseline (BL) and these terms are additive to the first harmonic of the absorbance *H*_1_. Generally, the spectrum of the noise occupies the band up to several tens of kHz. Using DL modulation at frequencies above hundreds of kHz and lock-in detection, one can separate the first harmonic of the signal *H*_1_ from the low-frequency noise of the $\ln{\left(\frac{\tau_{r}}{\tau_{s}}\right)}_{1f}$. The interference term $\ln{\left(\frac{\tau_{\nu r}}{\tau_{\nu s}}\right)}_{1f}$ can be reduced by optimization of the optical scheme of the sensor and by the appropriate processing. As a result, by slow scanning of the DL around the absorption line and by modulation of the DL current with a frequency *f*, we can detect the first harmonic of the absorption signal on a slow varying baseline. Knowing the experimental spectrum *U_out,_*_1*f*_ (*ν*), one can obtain the integral intensity *A* of the absorption line by fitting the simulated $H_{1}\left(\nu \right)$ spectrum to the measured one using the non-linear least squares method. The integral intensity *A*, linewidth Δ*ν*, modulation amplitude *a_m_*, and position *ν*_0_ of the line center can be used as free parameters. 

The noise reduction in our scheme differs from the scheme used in WMS-*nf*/1*f*. In the *nf*/1*f* technique, optical noises are reduced by normalization of a signal of an *n*-*th* harmonic (in particular 2*f*) to the signal of 1*f* harmonic, while on the beam path both signals gain the same optical noise components. In our scheme, conversion of the multiplicative components of the currents of the signal and reference channels are converted into the additive ones in LC. Equal components in both channels are canceled by subtraction of the reference current from the signal one. The fluctuating noise components of the signal gained on the optical path due to the turbulences are separated from the component of the selective absorption in the LIA. Such separation is based on (1) the additive character of the terms, and on (2) the frequency filtration of the signal from noises in the spectral range below frequency of the first harmonic. Thus, the proposed technique reduces optical noises and provides the realization of calibration-free measurements of line parameters as in the widely used scanned DAS and WMS *nf*/1*f* methods.

### 3.2. Fitting of the 1f-WMS Spectra

**Figure 2:**
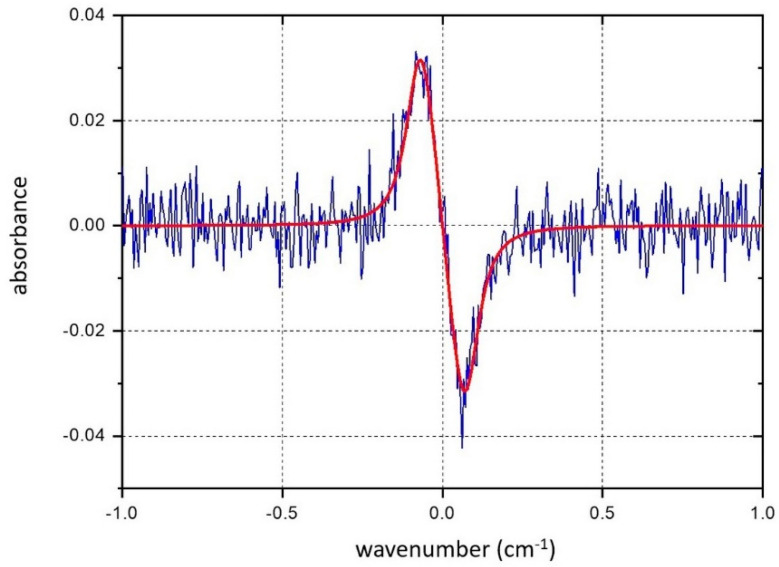
Fitting of a single realization of the theoretically constructed $H_{1}\left(\nu \right)$ spectrum of an absorption line with a mean white noise density of ~15% of the line amplitude.

The algorithm of the 1*f* harmonic fitting is the same as used in [26,36]. In the first step, the $H_{1}\left(\nu \right)\;$spectrum of an absorption line was constructed assuming a Voigt profile with a Lorentzian half-width at half-maximum (HWHM) Δ*ν*_L_ = 0.05 cm^−1^, the Doppler HWHM Δ*ν*_D_ = 0.0104 cm^−1^, and integral line intensity *A* = 0.01 cm^−1^. The linewidth used was close to the actual experimental value of the water absorption line at *ν*_0_ = 7185.6 cm^−1^. The white noise with a mean noise density of ~15% of the line amplitude was added to the $H_{1}\left(\nu \right)$ spectrum. This noisy spectrum was treated as the “experimental” one. In the next step, this “experimental” spectrum was fitted by the theoretically simulated one; 100 random realizations of a noisy “experimental spectrum” were processed using the proposed algorithm. Typical results of fitting of a single realization are shown in Figure 2.

The fitting parameters were the integral line intensity *A*, the modulation amplitude *a_m_*, displacement of the absorption line center δ*ν*_0_, and Lorentzian HWHM Δ*ν*_L_. Table 1 presents the results of fitting: the mean values of the appropriate parameters and their standard deviations *σ*. Four versions of the fitting procedure are presented: line 2—all four parameters are varied, line 3—modulation amplitude *a_m_* is fixed, line 4—Lorentzian half-width Δ*ν*_L_ is fixed, line 5—only the integral intensity *A* is varied. The results are the mean values of 100 random realizations.

Precise knowledge of the linewidth Δ*ν*_L_ noticeably reduces the error *σ*. Comparison of the values in columns 4 and 5 shows that variations in all four parameters provide reasonable precision for the value *a_m_*. However, precise knowledge of the modulation amplitude *a_m_* improves the precision of the evaluation of the integral intensity *A* from 0.000092 to 0.000062. The same is true for precise knowledge of the linewidth. The exact value of Δ*ν*_L_ improves the estimation of *A* from 0.000092 to 0.0000034.

## 4. Results and Discussions

### 4.1. Efficiency of the MPRR

A cheap commercial MPRR (3M diamond-grade micro-prismatic reflective sheeting 4090) designed for traffic safety was used in the developed sensor. The MPRR is actually a mosaic matrix that consists of separate rhombic sections of 4 × 4 mm in size. Each section consists of micro-prisms of approximately 120 × 120 μm in size. The sections are separated by the thin plane strips that act as dead zones for the reflected light. Similar dead zones are located at the prisms’ edges. However, the shapes of the fabricated micro-prisms are not precise, thus the reflected beam, which is initially parallel, diverges slightly. Because of this, the probing DL beam was slightly focused onto the MPRR to obtain maximum collection efficiency of the beam reflected from the MPRR onto the input of the fiber. It was found experimentally that a beam diameter of 2 mm on the MPRR surface was optimal. Such focusing provides a photocurrent in the signal channel of about 200 μA. This corresponds to an efficiency of the DL beam collection of about 1%. Tougher focusing onto a 1 mm diameter resulted in an increase in the collection efficiency of up to 6%, but at the expense of a dramatic increase in fluctuations of the signal intensity caused by the random displacement of the position of the focused beam on the surface of the MPRR due to the acoustic noise. 

The MPRR is the critical element of this scheme. A large part of the transmitted probe beam of the DL is reflected by the MPRR back to the multimode fiber, and about 1% of this reflected beam enters the fiber and is delivered to the detector. Only a negligible portion of the broadband thermal radiation of the hot zone enters the fiber. This allows for the term *E*(*t*) in Equation (1) to be canceled. The MPRR works as an efficient space filter. This is extremely important in probing the hot zones of real engines or propulsion devices. The MPRR simplifies the alignment of the optical scheme and increases the tolerance of the scheme to the vibration and deviations of the DL beam caused by the acoustic vibrations and turbulences in the probing zone. Lastly, the optical path of the beam in the probing zone is doubled. 

A scheme for sensors based on the backscattering of a probe beam has been reported in several publications [37,38,39,40,41,42]. Scattering from a native surface was reported in [38], and from the surfaces embedded into probes in [39]. The WMS of iodine vapor with the collection of backscattered laser radiation was reported in [40]. Detection of methane by WMS was reported in [41]. The WMS sensor with off-axis back reflection has been used for detection of the H_2_O absorption line around 1350 nm [39]. The SNR ~400 was realized using the laser beam reflection from a rough surface. The efficiency of light collection in this scheme was as low as ~5 × 10^−4^. Fiber coupled two-color measurements of temperature and an H_2_O concentration in a propane flame using the DL working at around 1.4 μm was described in [42].

Two advantages of our scheme can be highlighted. Firstly, the collection efficiency of other referred schemes was significantly lower, which dictated the use of a large solid angle of the reflected beam collection and, hence, efficient collection of the thermal background radiation. The collection efficiency for our scheme was about 20 times higher. Secondly, the proposed optical scheme enabled the use of a multichannel scheme with several fibers for probing different wavelengths. The fibers can be collected in a bundle to deliver the radiation with different wavelengths from a DL unit of the sensor to a probed zone. The absence of any influence of the inter-fibers will be explained in Section 4.7.

### 4.2. Tolerance to the Nature of Photodetector

**Figure 3:**
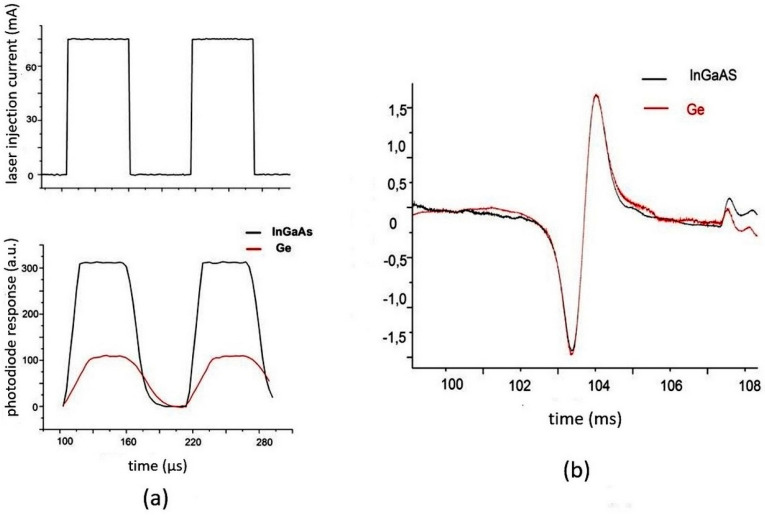
(**a**, **upper trace**) Meandric waveform of the DL injection current; (**a**, **lower trace**) output signals of InGaAs and Ge photodiodes without logarithmic detection; (**b**) 1*f* harmonic of the logarithmically converted signal of the 7185.6 cm^−1^ absorption line detected by the same detectors.

Equation (12) shows that the logarithm of the 1*f* signal does not depend on the parameters of the photodetector. This was investigated experimentally by the comparison of two quite different detectors—an InGaAs pin detector with a sensitive area of 2 mm (Hamamatsu) and a Ge pin detector with a sensitive area of 1 mm. The results are presented in Figure 3. Detection of the DL radiation by the two detectors modulated by meandric form is shown in Figure 3b. The large difference in the signals of the two detectors is evident. Figure 3a shows the signals to be proportional to the logarithms of a 1*f* harmonic of the absorption line at 7185.6 cm^−1^ detected by the same detectors. The peak-to-peak values of both signals differ by less than 1%. 

### 4.3. Tolerance to the Turbulences in the Optical Path

The integral line intensity was determined by logarithmic processing of the 1*f* harmonic of an absorption line, and tolerance of the proposed algorithm to the turbulences in the optical path of the DL probing beam was checked experimentally. The strong variation of the optical transmittance was modeled by inserting a plastic plate into the beam. The plate surface was roughly polished and could be rotated with a speed of 3000 rpm (see Figure 1). In this way, the transmittance of the optical path was randomly modulated. This method is similar to the modulation of the transmittance by a transverse supersonic jet used in [29].

**Figure 4:**
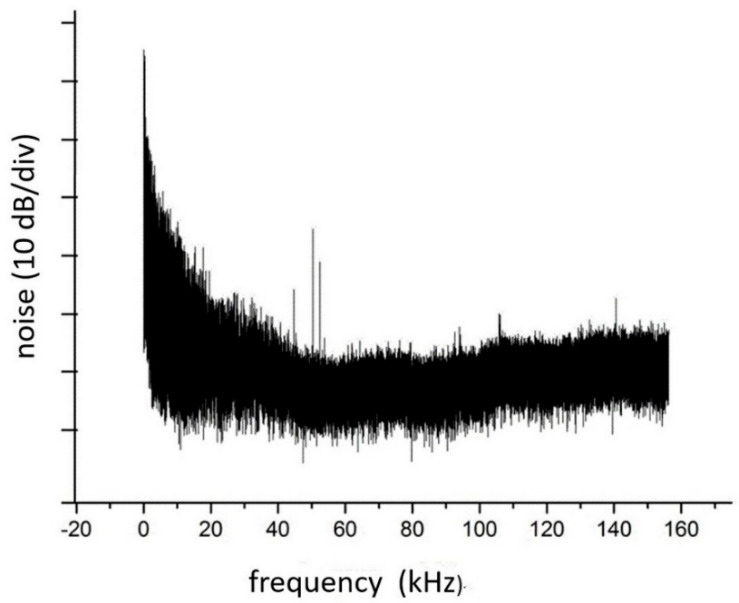
Noise spectrum of the photocurrent in the sample channel with rotating plastic plate in the optical path.

The DL intensity in these experiments was constant. The noise spectrum of the transmitted radiation is presented in Figure 4.

**Figure 5:**
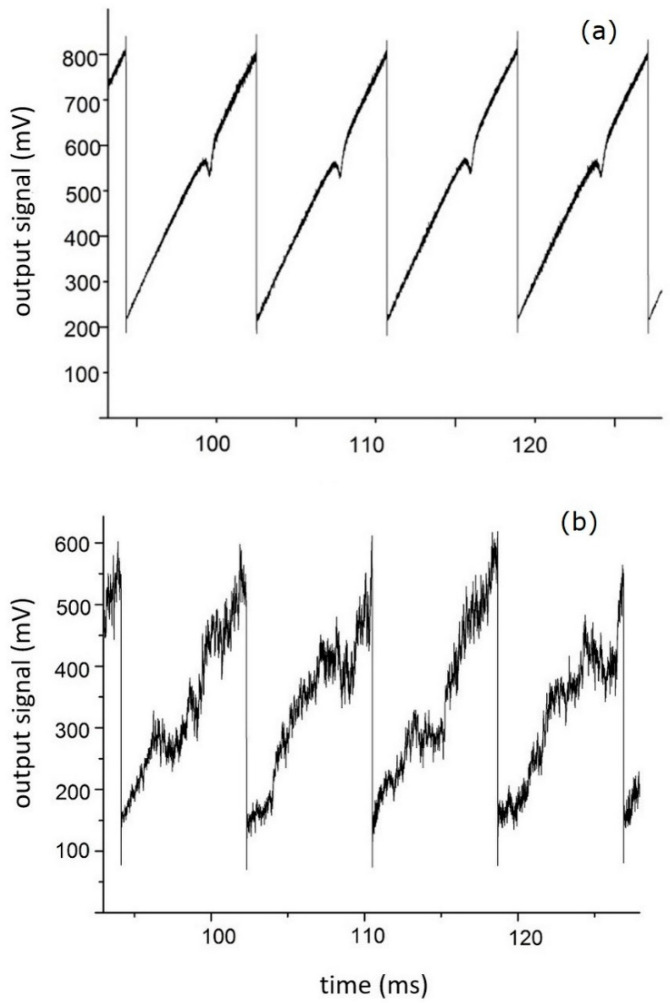
Four successive scans of the direct absorption signal recorded without (**a**) and with (**b**) beam distortion.

Figure 4 shows that the dominant type of noise of up to 50 kHz is flicker 1*f* noise. The influence of this noise on the direct absorption signal is shown in Figure 5. Four successive scans for the case when the plate is not inserted into the optical path are shown in Figure 5a, while four scans with the plate inserted into the optical path are shown in Figure 5b. The drastic effect of the “turbulence” in the optical path on the direct absorption signal is evident. There is no detectable signal in the latter case, and even when averaging over 100 scans an absorption signal cannot be extracted. 

**Figure 6:**
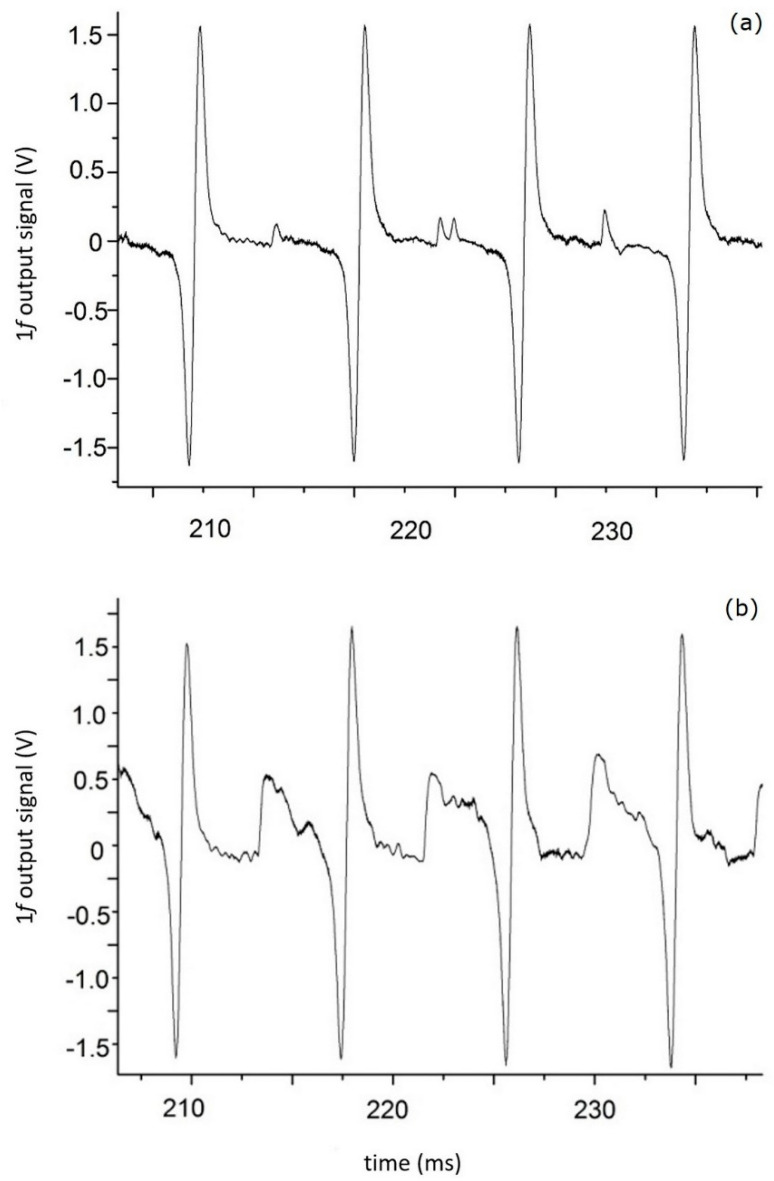
Four successive scans of the 1*f* signal recorded without (**a**) and with (**b**) beam distortion.

The efficiency of the logarithmic processing of the analytical signal is demonstrated in Figure 6. In these experiments, the DL radiation was modulated at 87 kHz and the first harmonic of the signal after logarithmic processing was detected.

**Figure 7:**
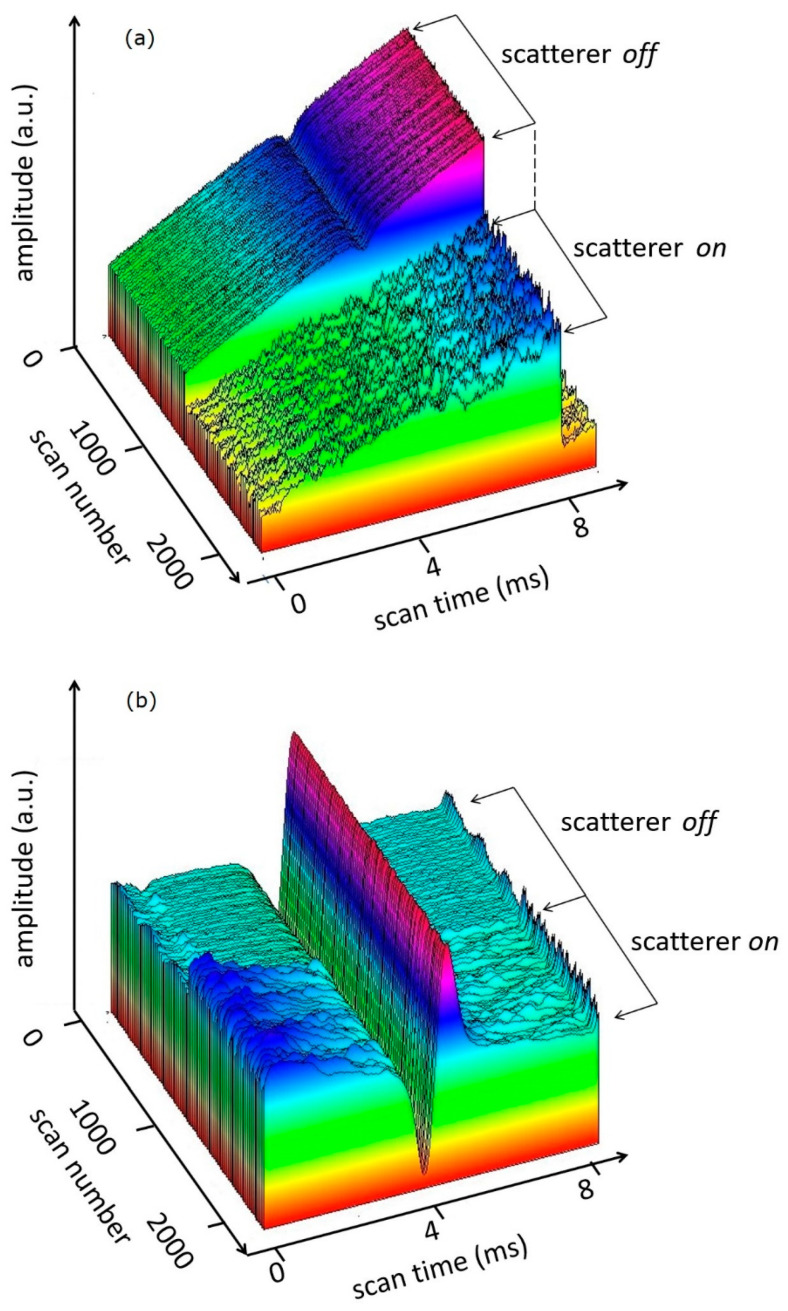
3D images of the direct linear signal (**a**) and the logarithmically converted 1*f* harmonic of the absorption signal (**b**) with and without the rotating plastic plate in the optical path.

Four successive scans of the first harmonic of the absorption line without the plastic plate in the optical path are shown in Figure 6a, while four scans with the plate inserted in the optical path are shown in Figure 6b. Despite the increase in the baseline in the latter case, the amplitude of 1*f* is practically unchanged. More evidence of the efficiency of the proposed algorithm is shown in Figure 7. 

Figure 7a presents 3D images of the logarithm of the 1*f* harmonic of the absorption signals for quiet and “harsh” environments. 

These experiments proved the independence of the analytical signal from the non-selective losses and their low frequency fluctuations.

### 4.4. Fitting of the Experimental Spectra

**Figure 8:**
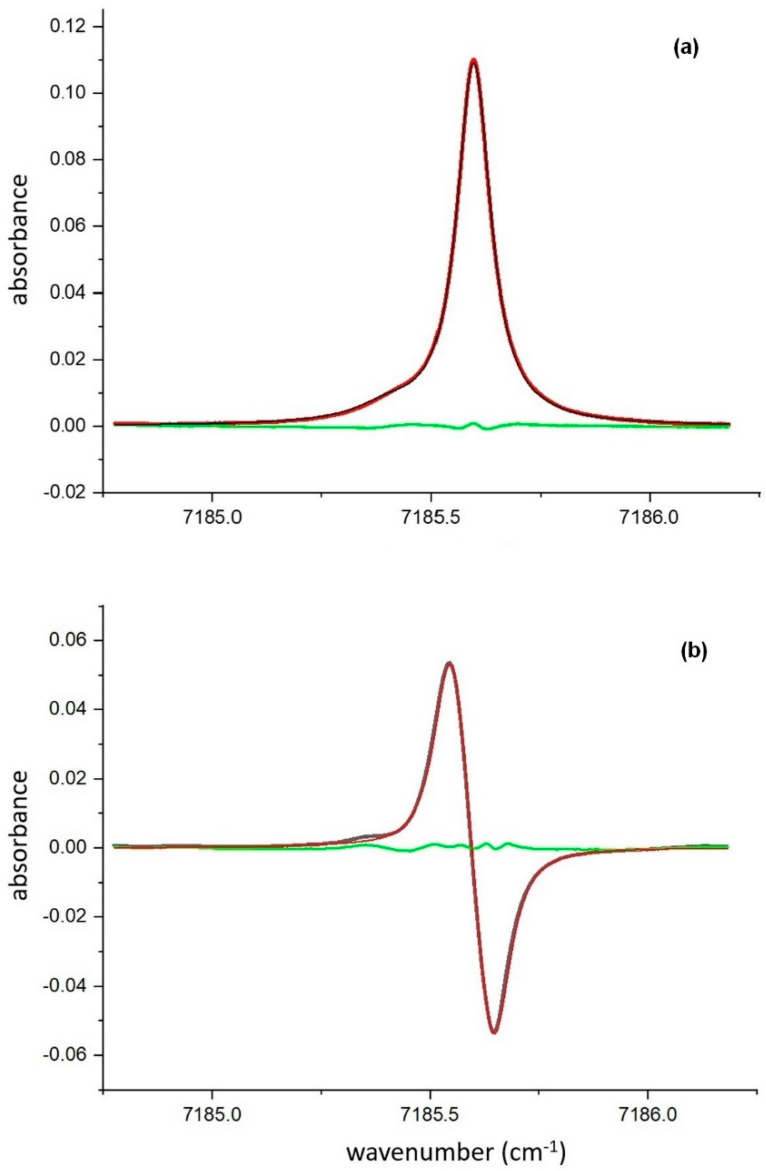
Results of fitting of the direct absorption (**a**) and log−1*f* harmonic (**b**) experimental signals of 7185. 6 cm^−1^ line. Residuals of both fitting procedures are shown in green.

In these experiments, the absorption line of a water molecule *ν*_0_ = 7185.6 cm^−1^ was used; the optical path was 80 cm, and the water vapor concentration was ~1% (absorbance ~0.11). Spectra of the direct absorption and the 1*f* harmonic are shown in Figure 8.

Both experimental spectra were fitted assuming the Voigt profile of the absorption line with the Doppler HWHM 0.0104 cm^−1^ and using the following variables: line intensity, Lorentzian linewidth, and position of the line center. For fitting the 1*f* harmonic, the modulation amplitude *a_m_* was added as a variable. 

The best fit for the direct absorption line was achieved with the following parameters: Lorentzian linewidth of 0.0429 cm^–1^ and integral line intensity of 0.0157 cm^−1^. The same parameters for the best fit of the 1*f* harmonic were: modulation amplitude of 0.056 cm^−1^, Lorentzian HWHM of 0.0436 cm^−1^, and the integral line intensity of 0.0150 cm^−1^. 

The estimated Lorentzian HWHM of the absorption line, determined by fitting the signal of direct absorption, and the signal of the 1*f* harmonic differ by less than 2%. The estimated integral line intensities for the direct absorption and the 1*f* harmonic differ by less than 5%. This validates the proposed technique for data processing.

### 4.5. Linearity

**Figure 9:**
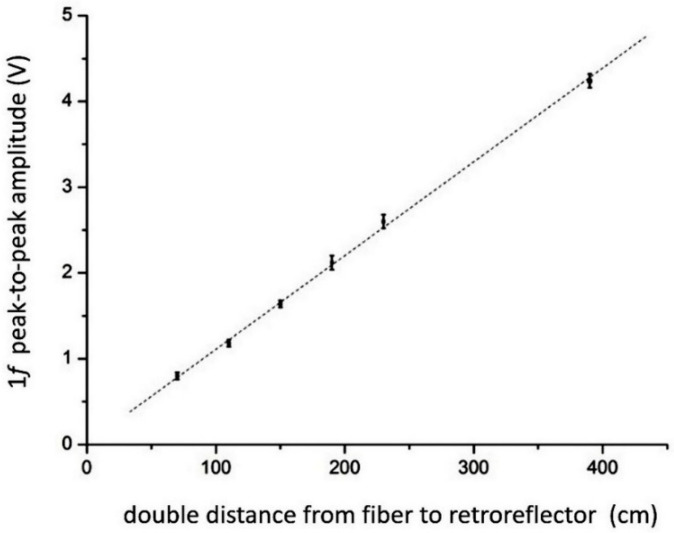
Dependence of the peak-to-peak amplitude of the 1*f* signal on the optical path length.

Linear dependence of the amplitude of the first harmonic on the optical density was checked experimentally. The water vapor concentration was fixed, and the optical density was varied by changing the position of the MPRR from the DL unit. The geometric lengths of the quartz tubes used as the cells varied from 30 cm to 2 m, which was physically equal to double the distance—up to 4 m—due to the MPRR. The same absorption line at 7185.6 cm^−1^ was used in the experiments. Figure 9 exhibits a linear dependence of the first harmonic amplitude on the optical length. 

### 4.6. Effect of Non-Ideal LC

Equation (12) predicts the BL even in the case of an ideal LC, which is defined by the 1*f* components of the interference and fluctuating non-selective transmissions within the frequency band of the LC. Real LC based on the *p*-*n* junction additionally contributes to the BL. Deviation from the ideal LC feature of the real LC is mostly defined by the specific parameters of a real transistor and by parasitic capacitors. The influence of these factors is treated in detail in the Appendix A. 

Thus, the real LC used in our experiments provided the difference in the phases of the sample and reference channels. In each channel, the phase shift *φ_pn_* between the input photocurrent and the *p*-*n* junction current can be written in the form:

(13) $$-\mathrm{tg}\varphi_{pn}=\frac{2\pi f\cdot C\cdot kT_{pn}}{q\cdot i_{pn}}$$

where *C* is the parasitic capacity of the *p*-*n* junction, *k* is the Boltzmann constant, *T_pn_* is the temperature of the junction, *q* is the electron charge, and *i_pn_* is the current through the *p*-*n* junction.

Following Equation (13), the phase shift *φ_pn_* depends on the *p*-*n* junction current. As shown in the Appendix A, a non-ideal feature of the LC produces the filtered output signal of the LIA in the form:

(14) $$U_{out,1f}=H_{1}\left(1-\theta_{1}^{2}\right)+\left(\theta_{1}^{2}-\varphi_{1}^{2}\right)X_{1}+\left(\varphi_{1}-\theta_{1}\right)Y_{1}$$

where *θ*_1_ and *φ*_1_ are the phase shifts of the first harmonic in the sample and reference channels, respectively.

In Equation (14), the first term is the first harmonic of the absorption coefficient *α*(*ν*) reduced by the coefficient $\left(1-\theta_{1}^{2}\right).\;$The second and third terms present the contribution of the non-ideal characteristics of a real LC in the BL. For small phase shifts, the quadratic terms in Equation (14) can be neglected and then:

(15) $$U_{out}\approx H_{1}+\left(\varphi_{1}-\theta_{1}\right)Y_{1}$$

If the photocurrents in the sample and reference channels are close to each other, the differences in the phase shifts are small and the BL is close to zero. The photocurrent in the reference channel is fixed, therefore a decrease in the sample photocurrent due to the attenuation in the absorption cell results in an increase in the phase difference which, in turn, increases the BL. If *i*_s_ << *i*_r_ then *φ*_1_ << *θ*_1_, and the BL is mostly defined by the term *θ*_1_*Y*, which, according to Equation (13), decreases with an increase in the DL injection current, namely during the DL scan. Such behavior of the BL is clearly seen in Figure 6b. Accordingly, in general, the fitting of the BL must be included in the fitting procedure. 

### 4.7. Wavelength Multiplexing with MPRR and Multifiber Bundle

In many applications, several DLs working with different wavelength ranges are used. A number of DLs are used if different test molecules are to be detected, or absorption lines of a test molecule are to be detected over different wavelength intervals, or the spatial profile for the temperature is to be obtained. Multi-wavelength problems can be solved either by time division multiplexing (TDM), frequency division multiplexing (FDM), or wavelength division multiplexing (WDM) schemes. With TDM, various DLs work in different successive time intervals and for de-multiplexing, the recording system detects the transmitted DL radiation in the correct time interval [43,44,45]. Alternatively, in FDM, radiation for the different DL is modulated with various frequencies and the transmitted radiation is demodulated and detected at the corresponding frequencies [46]. In the more sophisticated WDM version of a DLAS sensor, the beams of different DLs, after passing the probed volume, were spatially decoupled by dispersive optics (such as gratings) and directed to various photodetectors [47]. This system was more complex and less stable.

Our construction provides a new scheme for multi-wavelength probing. Radiation from different DLs propagates along a particular single-mode fiber, and different fibers are collected in a multi-mode bundle. The output radiation is focused by the lens into the probing volume (see Figure 1). The transmitted beam is reflected back by the MPRR and focused by the same lens at the output/input surface of the bundle. For high quality MPRR, practically all the radiation is reflected into the same individual single-mode fiber. The beams of the different DLs transmitted and reflected by the MPRR propagate along the particular fibers to the various photodetectors. If the distance between the axes of two neighboring fibers is 130 μm, the focal length of the lens used is 8.13 mm, and the angle between the beams from the neighboring fibers will be ~0.9 grad. For a distance of 400 mm from the bundle output surface to the probed volume, this angle provides a distance between the beams inside the volume of ~6 mm. For many applications this distance is not critical, and one can accept that beams will be testing the same hot zone. For a lens with a larger focal length, the distance between the neighboring beams from different DL will be even less. This construction allows simultaneous probing of a test volume at different wavelengths, while the modulation frequency can remain the same for all DLs.

In our experiments, three multimode fibers with 50 μm cores and 125 μm outer layers were combined in a 300 μm stainless-steel tube and bound together by an epoxy compound. The face of this bundle was polished. DL radiation with a wavelength of 1.39 μm was directed along the first fiber, which was inserted into the setup (see Figure 1). The 10 mW radiation from the second DL, with a wavelength 1.34 μm, was directed into the neighboring fiber. Both DLs were modulated with a frequency of 87 kHz. The interference between the DL radiation in the neighboring fibers was estimated by measuring the signal in the photodiode of the sample channel when the first DL was off and the second was at its maximal power. This interfering signal in the sample channel was less than 10^−4^ of the signal maximum in the sample channel. The same results were obtained with other pairs of fibers. Thus, three independent measurement channels can be created for probing virtually the same test volume and using the same modulation frequency of the DLs, which simplifies the sensor construction and data processing. 

## 5. Conclusions

A new scheme for a calibration-free DLAS sensor for measuring the parameters of harsh zones is proposed. The key element of the scheme is the micro-prism retroreflector (MPRR). This unit automatically reflects the probing laser beam back into a small core of optical fibers, and finally to the detector. The MPRR drastically reduces the broadband thermal radiation of a tested hot zone, performing as an efficient space filter. This optical scheme improves the mechanical stability of the sensor and its tolerance to the acoustic noise in the mechanism being tested (engines, propulsions, etc.) compared with mirror reflectors. As an additional bonus, the MPRR increases the effective total optical path within the hot zone two-fold.

Substantial reduction in the broadband thermal emission of the hot zones allowed a differential logarithmic conversion (LC) technique to be applied correctly for elimination of the residual amplitude modulation and other sources of non-selective attenuation of the probing laser beam. The LC permits direct detection of the absorption signal, and also the use of a 1*f*-WMS detection technique. The amplitude of the 1*f* harmonic is higher than the amplitudes of all other harmonics, which means higher sensitivity. It is also important that *H*_1_(*ν*) linearly depends on the optical path. Combination of LC and the 1*f*-WMS algorithm provided a new modification of the calibration-free DLAS. The developed scheme may be particularly useful for probing harsh zones with pronounced strong turbulence and high levels of acoustic and electrical noise. 

If a *p*-*n* junction is used as an LC, a phase shift between the sample and the reference channels can arise. This shift depends on the modulation frequency and amplitude of the photocurrents and can increase the baseline and thus decrease the absorption signal. This should be accounted for during the signal processing. 

The proposed optical scheme of a DLAS sensor and an algorithm for data processing provides a straightforward way of obtaining the integral intensity of an absorption line. In this paper, the potentials of the scheme were exemplified with a single water vapor absorption line at 7185.6 cm^−1^. 

Clearly, the developed algorithm for determination of the integral line intensities by processing the 1*f* harmonics of two experimentally detected absorption lines provides the final goal of a DLAS sensor—determination of the temperature and partial pressure of a test molecule in the probed gas volume. 

First experiments showed the lack of the interferences between the fibers (channels) in the bundle. This scheme potentially allows spatial multiplexing of the radiation of different DLs, which can be modulated with the same frequency, thus simplifying the construction of a sensor. Several DLs can be used if different test molecules need to be detected, or absorption lines of a test molecule are required at different wavelength intervals, or if the spatial profile for the temperature must be obtained.

## Figures and Tables

**Table 1:** Results of the fitting of the 1f spectrum of the absorption line (units for *A*, *a_m_*, δ*ν*_0_, Δ*ν*_L_, and *σ* are cm^−1^).

	*A*	*σ*(*A*)	*a_m_*	*σ*(*a_m_*)	δ*ν*_0_	*σ*(δ*ν*_0_)	Δ*ν*_L_	*σ*(Δ*ν*_L_)
4 parameters were varied	0.01011	0.000092	0.0792	0.00037	–0.0001	0.00016	0.05016	0.00046
*a_m_* fixed	0.00994	0.000062	0.08	–	–0.0001	0.00015	0.0496	0.00034
Δ*ν*_L_ fixed	0.010037	0.000034	0.0795	0.00028	–0.0001	0.00016	0.05	–
*a_m_*, Δ*ν*_L_, δ*ν*_0_ were fixed	0.009999	0.000031	0.08	–	–	–	0.05	–

## Appendix A

In Equation (8a,b) the LC coefficients and phase shifts in the sample and reference channels were assumed to be equal. Deviation of the real LC characteristics from the ideal ones are mostly defined by the non-ideal characteristics of the transistors (current gain non-linearity, bulk resistance) and by the parasitic capacitances. More detailed analysis of these factors is considered below.

The MAT-04 transistors have a typical bulk emitter resistance of *R_bulc_* = 0.4 Ω and a permanent current gain of h = 600 within a collector current interval of 10–1000 µA. In an ideal *p*-*n* junction, voltage *V* and junction current *i_pn_* are connected in the form:

(A1) $$i_{pn}=i_{0}\left[\exp(-\frac{qV}{kT_{pn}}\right)-1]$$

where *i*_0_ is the leakage current, *q* is the electron charge, *k* is the Boltzmann constant, and *T_pn_* is the temperature of the junction.

Small variations in the voltage at the ideal *p*-*n* junction caused by variation of the current *Δi* can be expressed as:

(A2) $$\Delta V=\frac{kT_{pn}}{q}\cdot \frac{\Delta i}{i}$$

Volume resistivity provides the addition voltage:

(A3) $$\Delta V=R_{bulc}i\cdot \frac{\Delta i}{i}$$

For a junction temperature of ~300 K and a photocurrent of less than 60 µA, the linear contribution of the bulk resistance (A3) becomes less than 10^−3^ of the logarithmic contribution (A2). In our differential recording scheme, this contribution to the output signal is even lower due to balancing of the currents in the signal and reference channels. Thus, for currents below 60 µA, the deformation of the logarithmic conversion is negligible.

The parasitic capacitance, C, of the *p*-*n* junction forms the divider for the alternative current. It causes a decrease in the amplitude and a phase shift of the alternative component of the photocurrent through the junction, which provides the logarithmic conversion. The effective parasitic capacitance consists of the intrinsic capacity of *p*-*n* junction, the input capacity of the operational amplifier, and the bootstrapped capacity of the photodiode, and can exceed 100 pF. Taking into consideration that the dynamic resistivity of the *p*-*n* junction is $\frac{kT_{pn}}{qi_{pn}}$, one can estimate the phase shift φ between the input photocurrent and the current through the *p*-*n* junction as:

(A4) $$-\mathrm{tg}\varphi =\frac{2\pi f\cdot C\cdot kT_{pn}}{q\cdot i_{pn}}$$

For a modulation frequency of 87 kHz, capacity 100 pF, and current 60 µA, the phase shift is low: tg*φ* ≈ sin*φ* ≈ *φ* ≈ 2 × 10^−2^. In this case, the decrease in the amplitude of the alternative current through the junction is equal to:

(A5) $$\cos\varphi \approx 1-\frac{\varphi^{2}}{2}\approx 1$$

Shunting of a *p*-*n* junction by the parasitic capacitances demands the correction of the logarithmic conversion coefficient and output signal of the LIA. Expansion of Equation (8a,b) in the Fourier series results in:

(A6) $$\ln[I_{slow}+a\cos\left(2\pi ft+\psi_{1}\right)+b\cos\left(4\pi ft+\psi_{2}\right)]=\overset{\infty }{\underset{0}{\sum }}(X_{n}\cos2\pi nft+Y_{n}\sin2\pi nft)$$

where *Xn* and *Yn* are the coefficients of expansion. Shunting of the *p*-*n* junctions of the reference and signal channels by the parasitic capacitances results in an increase in the phase shifts in each harmonic of the Fourier series, and in a decrease in the corresponding amplitudes (factor cos of the corresponding angle). Taking this into account, the voltage on the real LC of the reference channel can be written in the form:

(A7) $$\frac{U_{r}\left(real\right)}{K}=\ln\tau_{r}+\overset{\infty }{\underset{0}{\sum }}[X_{n}\cdot \cos\phi_{n}\cdot \cos(2\pi nft+\phi_{n})+Y_{n}\cdot \cos\phi_{n}\cdot \sin(2\pi nft+\phi_{n})$$

The same value for the sample channel with respect to Equation (10) is:

(A8) $$\frac{U_{s}\left(real\right)}{K}=\ln\tau_{s}+\overset{\infty }{\underset{0}{\sum }}[X_{n}\cdot \cos\theta_{n}\cdot \cos(2\pi nft+\theta_{n})+Y_{n}\cdot \cos\theta_{n}\cdot \sin(2\pi nft+\theta_{n})-\overset{\infty }{\underset{0}{\sum }}H_{n}\cdot \cos\theta_{n}\cos(2n\pi ft+\theta_{n})$$

where *φ_n_* is the phase shift of the *n*-*th* harmonic in the reference channel and *θn* is the phase shift of the *n*-*th* harmonic in the sample channel. After trigonometric transformation (taking into account $\phi_{n},\theta_{n}\;$<< 1 and assuming *K* = 1) the differential voltage in the real LC takes the form:

(A9) $$U_{out}\left(real\right)=U_{r}\left(real\right)-U_{s}\left(real\right)\approx (\ln\tau_{r}/\tau_{s})+\alpha +\overset{\infty }{\underset{0}{\sum }}[\left(\theta_{n}^{2}-\phi_{n}^{2}\right)X_{n}-\theta_{n}^{2}H_{n}+\left(\phi_{n}-\theta_{n}\right)Y_{n}]\cos2\pi nft+\overset{\infty }{\underset{0}{\sum }}[(\theta_{n}^{2}-\phi_{n}^{2})Y_{n}-\theta_{n}H_{n}+\left(\theta_{n}-\phi_{n}\right)X_{n}]\sin2\pi nft$$

Phases *φ_n_* and *θ_n_* depend on the photocurrent and, hence, oscillate. For small modulation amplitudes, one can neglect these oscillations and only take into account the slow changes of the phases, which are defined by the increase in the injection current during the DL scanning. If the LIA is tuned to the first harmonic and its phase coincides with the phase of the optical frequency modulation, then terms with sin in Equations (A7) and (A8) can be omitted. In such a case, the output signal of the LIA, after filtration, takes the form:

(A10) $$U_{out,1f}\approx H_{1}\left(1-\theta_{1}^{2}\right)+\left(\theta_{1}^{2}-\phi_{1}^{2}\right)X_{1}+\left(\phi_{1}-\theta_{1}\right)Y_{1}+{\left(\ln\tau_{r}/\tau_{s}\right)}_{1f}$$

Contrary to the ideal LC, in real LC a BL appears, which depends on the phase shifts in the reference and sample channels, and also on the amplitude of the decreases in the absorption signal.

The first term in Equation (A10) is the first harmonic of absorbance α reduced by the coefficient (1 − *θ*_1_^2^). The second and third terms define the contributions of a real LC to the BL. For small phase shifts, the quadratic terms in Equation (A10) can be canceled and then the expression for the output signal of the LIA takes the form:

(A11) $$U_{out,1f}\approx H_{1}\left(1-\theta_{1}^{2}\right)+\left(\varphi_{1}-\theta_{1}\right)Y_{1}$$

If the photocurrents in the signal and reference channels are close to each other, the difference of the phase shifts is slight, and the BL is close to zero. The current in the reference channel is fixed, so if the photocurrent in the signal channel reduces because of the absorption and scattering of DL radiation in the tested medium, then the difference in the phase shifts increases, which causes an increase in the BL. If *i_s_ << i_r_* then *φ_1_* << *θ_1_* and the BL is defined by the term *θ_1_Y_1_*, which reduces with increasing DL intensity, see Equation (A4). The DL intensity increases during the scan and the pointed effect is seen clearly in Figure 6b. Thus, fitting of the BL must be included in the general scheme of the fitting procedure.

## References

[1] Werle P., Slemr F., Maurer K., Kormann R., Mücke R., Jänker B. (2002). Near- and mid-infrared laser-optical sensors for gas analysis. Opt. Lasers Eng..

[2] Song K., Jung E.C. (2003). Recent Developments in Modulation Spectroscopy for Trace Gas Detection Using Tunable Diode Lasers. Appl. Spectrosc. Rev..

[3] Lackner M. (2007). Tunable Diode Laser Absorption Spectroscopy (TDLAS) in the Process Industries—A Review. Rev. Chem. Eng..

[4] Mantz A.W., Nadezhdinskii A.I., Sigrist M.W., Tittel F.K. (2010). Special issue “7th International Conference on Tunable Diode Laser Spectroscopy. ” Appl. Phys. B.

[5] Hodgkinson J., Tatam R.P. (2013). Optical gas sensing: A review. Meas. Sci. Technol..

[6] Rodin A., Klimchuk A., Nadezhdinskiy A., Churbanov D., Spiridonov M. (2014). High resolution heterodyne spectroscopy of the atmospheric methane NIR absorption. Opt. Express.

[7] Wang F., Jia S., Wang Y., Tang Z. (2019). Recent Developments in Modulation Spectroscopy for Methane Detection Based on Tunable Diode Laser. Appl. Sci..

[8] Allen M.G. (1998). Diode laser absorption sensors for gas-dynamic and combustion flows. Meas. Sci. Technol..

[9] Bolshov M.A., Kuritsyn Y.A., Romanovskii Y.V. (2015). Tunable diode laser spectroscopy as a technique for combustion diagnostics. Spectrochim. Acta Part B At. Spectrosc..

[10] Goldenstein C.S., Spearrin R.M., Jeffries J.B., Hanson R.K. (2017). Infrared laser-absorption sensing for combustion gases. Prog. Energy Combust. Sci..

[11] Liu C., Xu L. (2018). Laser absorption spectroscopy for combustion diagnosis in reactive flows: A review. Appl. Spectrosc. Rev..

[12] Furlong E.R., Mihalcea R.M., Webber M.E., Baer D.S., Hanson R.K. (1999). Diode-Laser Sensors for Real-Time Control of Pulsed Combustion Systems. AIAA J..

[13] Bolshov M.A., Kuritsyn Y.A., Liger V.V., Mironenko V.R., Nadezhdinskii A.I., Ponurovskii Y.Y., Leonov S.B., Yarantsev D.A. (2015). Measurement of transient gas flow parameters by diode laser absorption spectroscopy. Quantum Electron..

[14] Witzel O., Klein A., Meffert C., Schulz C., Kaiser S.A., Ebert V. (2015). Calibration-free, high-speed, in-cylinder laser absorption sensor for cycle-resolved, absolute H2O measurements in a production IC engine. Proc. Combust. Inst..

[15] Nadezhdinskii A.I., Ponurovskii Y.Y. (2019). Diode Laser Spectrometer for High-Precision Measurements. Quantum Electron..

[16] Reid J., Labrie D. (1981). Second-harmonic detection with tunable diode lasers—Comparison of experiment and theory. Appl. Phys. B.

[17] Philippe L.C., Hanson R.K. (1993). Laser diode wavelength-modulation spectroscopy for simultaneous measurement of temperature, pressure, and velocity in shock-heated oxygen flows. Appl. Opt..

[18] Groll H., Schnürer-Patschan C., Kuritsyn Y.A., Niemax K. (1994). Wavelength modulation diode laser atomic absorption spectrometry in analytical flames. Spectrochim. Acta Part B At. Spectrosc..

[19] Linnerud I., Kaspersen P., Jaeger T. (1998). Gas monitoring in the process industry using diode laser spectroscopy. Appl. Phys. B Lasers Opt..

[20] Kluczynski P., Axner O. (1999). Theoretical Description Based on Fourier Analysis of Wavelength-Modulation Spectrometry in Terms of Analytical and Background Signals. Appl. Opt..

[21] Kluczynski P., Gustafsson J., Lindberg Å.M., Axner O. (2001). Wavelength modulation absorption spectrometry—An extensive scrutiny of the generation of signals. Spectrochim. Acta Part B At. Spectrosc..

[22] Cassidy D.T., Reid J. (1982). Atmospheric pressure monitoring of trace gases using tunable diode lasers. Appl. Opt..

[23] Duffin K., McGettrick A.J., Johnstone W., Stewart G., Moodie D.G. (2007). Tunable Diode-Laser Spectroscopy with wavelength modulation: A calibration-free approach to the recovery of absolute gas absorption line shapes. J. Light. Technol..

[24] Rieker G.B., Jeffries J.B., Hanson R.K. (2009). Calibration-free wavelength-modulation spectroscopy for measurements of gas temperature and concentration in harsh environments. Appl. Opt..

[25] Lan L.J., Ding Y.J., Peng Z.M., Du Y.J., Liu Y.F. (2014). Calibration-free wavelength modulation for gas sensing in tunable diode laser absorption spectroscopy. Appl. Phys. B.

[26] Goldenstein C.S., Strand C.L., Schultz I.a., Sun K., Jeffries J.B., Hanson R.K. (2014). Fitting of calibration-free scanned-wavelength-modulation spectroscopy spectra for determination of gas properties and absorption lineshapes. Appl. Opt..

[27] Upadhyay A., Lengden M., Wilson D., Humphries G.S., Crayford A.P., Pugh D.G., Johnson M.P., Stewart G., Johnstone W. (2018). A New RAM Normalized 1f-WMS Technique for the Measurement of Gas Parameters in Harsh Environments and a Comparison With2f/1f. IEEE Photonics J..

[28] Ruxton K., Chakraborty A.L., Johnstone W., Lengden M., Stewart G., Duffin K. (2010). Tunable Diode Laser Spectroscopy with Wavelength Modulation: Elimination of Residual Amplitude Modulation in a Phasor Decomposition Approach. Sens. Actuators B Chem..

[29] Peng W.Y., Strand C.L., Hanson R.K. (2020). Analysis of Laser Absorption Gas Sensors Employing Scanned-Wavelength Modulation Spectroscopy with 1f-Phase Detection. Appl. Phys. B Lasers Opt..

[30] Liger V.V., Kuritsyn Y.A., Krivtsun V.M., Snegirev E.P., Kononov A.N. (1997). Measurement of the absorption with a diode laser characterised by a detection threshold governed by the shot noise of its radiation. Quantum Electron..

[31] Liger V., Zybin A., Kuritsyn Y., Niemax K. (1997). Diode-Laser Atomic-Absorption Spectrometry by the Double-Beam—Double-Modulation Technique. Spectrochim. Acta Part B At. Spectrosc..

[32] Zybin A.V., Liger V.V., Kuritsyn Y.A. (1999). Dynamic Range Improvement and Background Correction in Diode Laser Atomic Absorption Spectrometry. Spectrochim. Acta Part B At. Spectrosc..

[33] Wang Y., Cai H., Geng J., Fang Z. (2009). Logarithmic Conversion of Absorption Detection in Wavelength Modulation Spectroscopy with a Current-Modulated Diode Laser. Appl. Opt..

[34] Cong M., Sun D. (2018). Detection of Ammonia Using Logarithmic-Transformed Wavelength Modulation Spectrum. IOP Conf. Ser. Mater. Sci. Eng..

[35] Bolshov M.A., Kuritsyn Y.A., Liger V.V., Mironenko V.R., Leonov S.B., Yarantsev D.A. (2010). Measurements of the temperature and water vapor concentration in a hot zone by tunable diode laser absorption spectrometry. Appl. Phys. B.

[36] Mironenko V.R., Kuritsyn Y.A., Liger V.V., Bolshov M.A. (2018). Data Processing Algorithm for Diagnostics of Combustion Using Diode Laser Absorption Spectrometry. Appl. Spectrosc..

[37] Li J., Yu Z., Du Z., Ji Y., Liu C. (2020). Standoff Chemical Detection Using Laser Absorption Spectroscopy: A Review. Remote Sens..

[38] Wang Z., Sanders S.T. (2015). Toward single-ended absorption spectroscopy probes based on backscattering from rough surfaces: H2O vapor measurements near 1350 nm. Appl. Phys. B.

[39] Smith C.H., Goldenstein C.S., Hanson R.K. (2014). A scanned-wavelength-modulation absorption-spectroscopy sensor for temperature and H_2_O in low-pressure flames. Meas. Sci. Technol..

[40] Dubinsky I., Rybak K., Steinfeld J.I., Field R.W. (1998). Frequency-modulation-enhanced remote sensing. Appl. Phys. B Lasers Opt..

[41] Wainner R.T., Green B.D., Allen M.G., White M.A., Stafford-Evans J., Naper R. (2002). Handheld, battery-powered near-IR TDL sensor for stand-off detection of gas and vapor plumes. Appl. Phys. B Lasers Opt..

[42] Goldenstein C.S., Mitchell Spearrin R., Hanson R.K. (2016). Fiber-coupled diode-laser sensors for calibration-free stand-off measurements of gas temperature, pressure, and composition. Appl. Opt..

[43] Liger V.V., Mironenko V.R., Kuritsyn Y.A., Bolshov M.A. (2019). Diagnostics of Hot Zones by Absorption Spectroscopy with Diode Lasers (Review). Opt. Spectrosc..

[44] Klein A., Witzel O., Ebert V. (2014). Rapid, Time-Division Multiplexed, Direct Absorption- and Wavelength Modulation-Spectroscopy. Sensors.

[45] Sur R., Sun K., Jeffries J.B., Socha J.G., Hanson R.K. (2015). Scanned-wavelength-modulation-spectroscopy sensor for CO, CO_2_, CH_4_ and H_2_O in a high-pressure engineering-scale transport-reactor coal gasifier. Fuel.

[46] Wei W., Peng W.Y., Wang Y., Shao J., Strand C.L., Hanson R.K. (2020). Two-color frequency-multiplexed IMS technique for gas thermometry at elevated pressures. Appl. Phys. B.

[47] Liu J.T.C., Rieker G.B., Jeffries J.B., Gruber M.R., Carter C.D., Mathur T., Hanson R.K. (2005). Near-infrared diode laser absorption diagnostic for temperature and water vapor in a scramjet combustor. Appl. Opt..

